# A Review of Breast Imaging for Timely Diagnosis of Disease

**DOI:** 10.3390/ijerph18115509

**Published:** 2021-05-21

**Authors:** Giulia Bicchierai, Federica Di Naro, Diego De Benedetto, Diletta Cozzi, Silvia Pradella, Vittorio Miele, Jacopo Nori

**Affiliations:** 1 Diagnostic Senology Unit, Azienda Ospedaliero-Universitaria Careggi, 50139 Florence, Italy; dinarof@aou-careggi.toscana.it (F.D.N.); diegodebenedetto1@gmail.com (D.D.B.); jakopo@tin.it (J.N.); 2 Emergency Radiology Department, Azienda Ospedaliero-Universitaria Careggi, 50139 Florence, Italy; cozzid@aou-careggi.toscana.it (D.C.); pradellas@aou-careggi.toscana.it (S.P.); vmiele@sirm.org (V.M.); 3 Foundation SIRM, 20122 Milan, Italy

**Keywords:** breast, mammography, ultrasound, tomosynthesis, MRI, CEM, breast cancer

## Abstract

Breast cancer (BC) is the cancer with the highest incidence in women in the world. In this last period, the COVID-19 pandemic has caused in many cases a drastic reduction of routine breast imaging activity due to the combination of various factors. The survival of BC is directly proportional to the earliness of diagnosis, and especially during this period, it is at least fundamental to remember that a diagnostic delay of even just three months could affect BC outcomes. In this article we will review the state of the art of breast imaging, starting from morphological imaging, i.e., mammography, tomosynthesis, ultrasound and magnetic resonance imaging and contrast-enhanced mammography, and their most recent evolutions; and ending with functional images, i.e., magnetic resonance imaging and contrast enhanced mammography.

## 1. Introduction

Breast cancer (BC) is the cancer with the highest incidence in women in the world. Last year (2020), 2.2 million new cases of BC were expected to be diagnosed worldwide [1]. The estimated total BC-related deaths for the last year were 684,996, although the spread of the worldwide COVID-19 pandemic could alter these estimates. In fact, the COVID-19 pandemic has caused in many cases a drastic reduction of routine breast imaging activity due to a combination of various factors, including reduced hospital resources, the need for social distancing and lockdowns, as reported in the documents published by some scientific breast imaging societies [2,3].

The survival rates of BC are directly proportional to earliness of diagnosis, and early detection contributes to a decrease in specific BC mortality. European BC mortality rates declined from 17.9/100,000 in 2002 to 15.2 in 2012, and the predicted 2020 rate is 13.4/100,000. This favorable trend is due to the constant improvement in the management and therapy of BC, in which early diagnosis certainly plays a fundamental role.

In this period, it is fundamental to remember that a diagnostic delay of even three months could affect BC outcomes [3,4,5].

In this article we will review the state of the art of breast imaging that allows increasingly early diagnoses and increasingly conservative treatments.

## 2. Morphological Imaging

The practice of breast imaging has seen the development a wide variety of technological advances from the early days to the current era, transitioning through direct-exposure film mammography and xeromammography, up to full-field digital mammography and digital breast tomosynthesis.

Ultrasonography is also complementary to mammography, and these imaging modalities have helped to shape the specialty of breast imaging. Together, ultrasound (US), mammography (DM) and tomosynthesis (DBT), with their most recent evolutions, make up the so-called morphological imaging, whose role remains fundamental in the early diagnosis of breast cancer [6].

## 3. Advent of Screening Mammography

In 1963, the first encouraging results of the Health Insurance Plan of Greater New York randomized clinical trial (HIP study) of screening mammography were published; this trial showed the advantages of film mammography through a significant reduction in breast cancer deaths [7].

Since then, at least eight randomized controlled trials (RCTs), have been performed and published, which show that mammography screening can reduce the breast cancer mortality by at least 20% [8,9].

In the late 1970s, two trials in Sweden, the Swedish Two-County trial and Malmö, investigated the effect of screening mammography without physical examination [10,11,12,13].

RCTs provide the strongest evidence for mortality reduction and have completely upset the epidemiology of breast cancer, whose prognosis was very poor before the introduction of mammography screening [14] Table 1 [10,11,13,14,15,16,17].

## 4. Digital Mammography

Digital mammography involves collecting the transmitted radiation on an electronic image detector instead of on film. Hence, the transition to digital mammography from film mammography was expensive for a breast imaging practice [18].

The Digital Mammographic Imaging Screening Trial was a landmark multi-institution study of the American College of Radiology Imaging Network comparing digital and film mammography in a screening population at 33 sites in the United States and Canada [19]. The study demonstrated overall equivalence between the two techniques. However, for women younger than 50; premenopausal and perimenopausal women; and women with heterogeneously dense or extremely dense breasts, digital mammography was more accurate than film mammography, and also for this reason, the transition from analogue to digital mammography systems began in the early 2000s.

By 1985, the use of screening mammography, initially carried out with film mammography and then from the 2000s more and more with the digital system, had grown considerably, demonstrating the effectiveness of mammographic screening against breast cancer, considered to be a major public health problem [20].

This awareness has led the American College of Radiology to begin its Breast Imaging Reporting and Data System (BI-RADS) to create a system for standardized reporting of mammography studies, in order to allow correct communication of findings and recommendations to the referring clinician [21,22]. BI-RADS was the first structured reporting language for imaging and contains three important components: a lexicon of descriptors; a reporting structure to include final assessment categories and management recommendations; and a framework for data collection and auditing [22].

Despite the surprising results of mammography screening on mortality reduction for breast cancer, data from various published studies highlighted a problem: about 50% of women undergoing screening mammography have dense breasts, and in dense breasts the sensitivity of mammography decreases, measuring 30% to 64% for extremely dense breasts (vs. 76% to 98% for fatty breasts) [23,24]. As dense tissue causes a “masking” phenomenon and obscures underlying cancers similarly to X-ray attenuation, women with dense breasts have a 1.2-fold to 2.1-fold higher risk of breast cancer, and the interval cancer rate is as much as 17-fold higher (a cancer diagnosed within 12 months of a negative screening mammogram) compared with women with the fattiest breasts [25]. Women with dense breast tissue constitute the largest population of “intermediate risk”—that is, women with a 15–25% lifetime risk of breast cancer [26].

The American College of Radiology (ACR) and Society of Breast Imaging recommend that women at average risk of breast cancer begin annual screening mammography at age 40 and stop screening when life expectancy is less than 5 to 7 years on the basis of age or a comorbid condition [27].

Carbonaro et al. [28] have evaluated that the estimate of the interval mammogram rate, i.e., the undertaking of an additional mammography between scheduled screening rounds, and identified factors influencing this phenomenon. This study showed a relatively low (15%) interval mammogram rate in women belonging to the local organized screening program with higher breast density, and the first rounds of program adherence were significantly associated with a higher interval mammogram rate.

To try to overcome the reduction of sensitivity of DM in dense breasts and to always improve early diagnosis, even in women at average risk of breast cancer, supplemental screening modalities have been investigated: digital breast tomosynthesis (DBT) and ultrasound (US) have now become fundamental techniques that integrate DM, particularly in the study of this subgroup of patients [25].

## 5. Digital Breast Tomosynthesis

**Figure 1:**
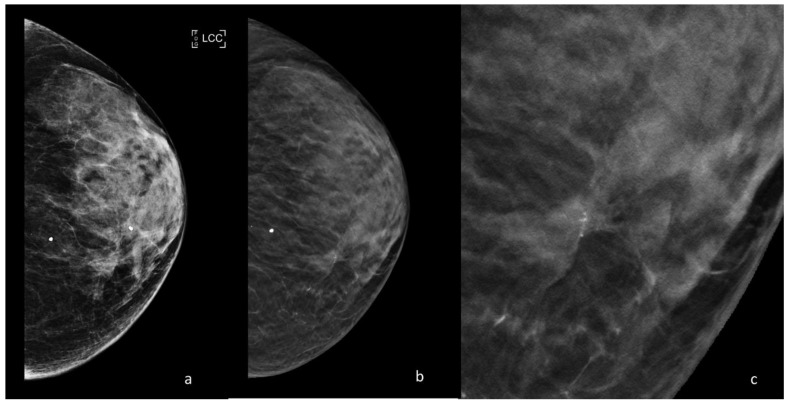
Periodic control with DM and DBT of a 47-year-old patient with a family history of breast cancer. (**a**) DM cranio-caudal left view; breast density BIRADS C; millimetric cluster of microcalcifications in the inner quadrants. (**b**) DBT cranio-caudal left view; in the site of the microcalcifications a parenchymal distortion is appreciated, enhanced by DBT acquisition. (**c**) Detail of the parenchymal distortion at higher magnification. The distortion with microcalcifications was then biopsied, resulting an invasive ductal carcinoma.

DBT is a digital mammogram technique that involves the acquisition of low-dose 2D X-ray projection images of the breast, as the X-ray tube pivots in an arc that varies between 15° (narrow range) and 60° (wide range) in a plane aligned with the chest wall. DBT also decreased the impact of overlapping breast tissue reducing false positives due to tissue summation and increasing conspicuity of occult lesions on DM, see Figure 1.

The dose of a single DBT view is slightly higher than the corresponding one-view DM (2.19 mGy vs. 1.88 mGy for 50–60 mm thick breasts) [29].

To reduce the radiation dose, the synthetic mammography (SM) has been developed resulting in a DM-equivalent image, in which two-dimensional images are reconstructed from the tomosynthesis dataset to replace the DM portion of the examination [30].

There is currently no standardization on whether and how to implement DBT in screening.

DBT has also been considered for use in combination with DM or in combination with SM, with SM replacing DM. Several studies have been performed comparing DM to DM with DBT [31].

Two major prospective clinical trials are the Oslo Tomosynthesis Screening Trial and the Tomosynthesis or Mammography (STORM) trial [31,32].

In the first trial, DBT with DM increased the invasive cancer detection rate by 40% and decreased false positives by 15%, compared with DM alone. In the STORM trial DBT increased the cancer detection rate from 5.3 to 8.1 for 1000 examinations with a simultaneous 17% reduction in recall rate. DBT has shown promising results compared to standard DM, but the costs of implementing the technology in screening programs are not yet known.

The increase in costs of equipment, examination and reading time with DBT vs. DM was €8.5 per screened woman (95% CI 8.4–8.6), and for the recall assessment cost it was €6.2 (95% CI 4.6–7.9) [33].

Some studies have shown that DBT allows an increase in the detection of invasive cancers rather than ductal carcinoma in situ, which, when lower grade, is sometimes considered as a harm of screening or “overdiagnosis” [18,22,34].

To further understand the impact of the increased detection of invasive cancers with DBT, Verona study, which included 315 cancers, demonstrated a higher proportion of cancer with histologic characteristics generally associated with a good prognosis (i.e., tubular, papillary and mucinous subtypes) compared with those detected with DM alone [35].

Therefore, at the moment there is no consensus on the introduction of DBT in screening, and we are awaiting studies that can better clarify the position of this technique, which, as we have seen, does allow for early diagnoses, especially in intermediate risk patients.

## 6. Ultrasound

Ultrasound (US) is a valid supplemental screening tool in women with dense breast tissue because it is widely available and low cost. The sensitivity of DM for the detection of breast cancer is reduced to 47.8–64.4% in patients with dense breasts, and bilateral screening US, using a high-frequency transducer, allows the detection of early stage mammographically occult breast cancers [36,37].

Available commercial systems use linear arrays operating at around 10–14 MHz with close to 100% bandwidth ranging from 5 to 18 MHz. Several studies showed the primary role of the US as a screening tool in women with dense breasts.

Berg et al. [38] have published the most significant multi-institutional trial, showing an increase in the diagnostic yield of breast cancer of 4.2 per 1000 women screened.

Other previous studies found that most cancers detected were invasive (91.7%), with a mean size of 10 mm, and the only limitation was an increase in biopsies compared to mammography alone [39]. However, the performance of bilateral handheld ultrasound makes it a challenge for screening, in terms of physician time for exam execution and interpretation (workflow of nearly 20 min).

Due to these limitations, automated breast ultrasound (ABUS) was introduced, a new ultrasonography technique with the purpose of overcoming the operator-dependence of handheld US, increasing the reproducibility of the examination [40].

Nowadays, two main categories of automated breast ultrasound systems are available: prone and supine scanners. Moreover, ABUS allows multiplanar reconstructions, especially the coronal view, also known as the “surgical view” (in which the breast is positioned in the same way that it is oriented on the surgical table), and it provides important information, such as the retraction phenomenon [41].

The coronal view allows significantly lower reading times and represents a valuable feature in the screening setting; diagnostic performance makes the complete multiplanar assessment mandatory. The main limitations of ABUS systems are the exclusion of axillary regions from the field of view and the absence of tools to assess vascularity and tissue elasticity [42].

The first screening work using ABUS was performed by Kelly et al. This multicenter study compared mammography alone versus automated whole breast ultrasound (AWBU) plus mammography in 4419 women with dense breasts and/or at elevated risk of breast cancer. They found an improvement in cancer detection of 3.6 per 1000 women screened with the addition of AWBU, and sensitivity increased from 40% for mammography alone to 81% for the combined modalities [43]. Of note, recalls increased from 4.2% for mammography alone to 9.6% adding AWBU.

An Italian review of Zanotel at al. [40] has evaluated the potential and limitations of ABUS as a method of choice and adjunctive tool to screening mammography in women with dense breast tissue.

Multiple studies have demonstrated similar sensitivities, cancer detection rates, diagnostic accuracy rates, and image quality for ABUS and US; however, ABUS had significantly longer execution times than US. The role of ABUS is still debated [44].

A further evolution is in the attempt to merge the ABUS and the DBT into a single device. The advantage of this new device is the ability to perform ABUS directly without decompressing the breast, in the same position in which the DBT is acquired. In this way the alterations identified with DBT could thus be better investigated without having to return the patient at a later time and without even having her move. These still initial experiences must be validated on a large scale, but from the first results the technique seems to be of sufficient quality to identify malignant lesions and could lead to important logistical and economic advantages, especially in the screening setting [45,46].

## 7. Elastosonography

Elastosonography has become a routine tool in ultrasonic diagnosis and measures the consistency or hardness of the tissues non-invasively by introducing mechanical excitation within a region of interest and measuring the induced disturbance, to differentiate benign from malignant breast lesions.

The induced disturbance is measured either as displacement within the field of view, and this is referred to as strain elastography (SE), or as the velocity of an induced shear wave, and this is referred to as shear wave elastography (SWE) [47].

Several single and multicentric studies have been performed to evaluate use of ultrasound for elastography in regard to the most recent guidelines for clinical use of elastography, published by World Federation of Ultrasound in Medicine and Biology (WFUMB), which recommend elastography for the characterization of solid breast masses [48].

The size of a mass influences the SWF result, and it has been reported that smaller lesions have better sensitivity and specificity [47].

When compared with conventional B-mode ultrasound, there was controversy regarding the accuracy of breast ultrasound elastography, and SWE was not significantly more sensitive than gray-scale ultrasound for the detection of either invasive ductal carcinoma or invasive lobular carcinoma [49].

Elastosonography, then, is a simple, fast and non-invasive diagnostic method that may improve the specificity of diagnosing breast cancer, especially for BI-RADS 3 lesions.

Elastography has been found to reduce the need for benign biopsies when they are used as a complementary tool to conventional US, and could then help to define the location for a biopsy and characterize a complex lesion [50].

Contrast-enhanced ultrasound (CEUS) can be used for characterizing masses of the breast, specifically, evaluating the differences in vascularity. Gas microbubbles encapsulated by an outer shell for stability are traditionally injected intravenously, and due to their size (<8 micrometer) are restricted to the vascular space. Insonated microbubbles are also highly nonlinear (through ultra-harmonic frequency components), which enable filtering approaches for separating microbubble echoes from surrounding tissue during imaging. Ricci et al. 2007 found that the sensitivity and specificity of CEUS for differentiating malignant from benign breast lesions are 100% and 87.5%, respectively, and contrast-enhanced sonographic patterns correlated well with those provided by MRI [51].

We await further studies on CEUS in order to understand the role of this new method in the early diagnosis of breast cancer.

In conclusion, the role of the morphological imaging has been fundamental to reducing the mortality of breast cancer and still maintains an essential role in early diagnosis of breast cancer, also thanks to the new technologies introduced that allow us to customize the screening for each patient.

## 8. Functional Breast Imaging

So-called functional breast imaging is essentially composed of contrast enhanced magnetic resonance imaging (ce-MRI) and the more recent contrast enhanced mammography (CEM). These two breast imaging techniques are based on the same principle, namely, “tumor neoangiogenesis.”

Tumor-induced neoangiogenesis causes the genesis of leaky vessels that allow for faster extravasation of intravenously injected contrast agents from the vessels towards the interstitium, thereby leading to rapid local enhancement. Ce-MRI evaluates the permeability of blood vessels by using an intravenous contrast agent (gadolinium chelate) that shortens the local T1 time, leading to a higher signal in T1-weighted images, whereas in CEM a non-ionic iodinated contrast agent is used which increases the absorption of X-rays in the tissues where it accumulates.

Functional imaging presents diagnostic performances clearly superior to conventional imaging, which will be analyzed in detail below (ce-MRI and CEM), and is today increasingly fundamental in clinical practice and must be well known by every radiologist that deals with the breast [52,53].

## 9. ce-MRI

Breast ce-MRI was introduced into clinical practice in the 1980s and is now widely used around the world. It is best practice to use a field strength of at least 1.5 T to acquire images at a sufficiently high spatial resolution and a dedicated breast coil with at least four channels (modern designs have 16 channels or more) to obtain diagnostic-quality images. The patient lies prone during the acquisition, which can have a variable duration depending on the study protocol used, from the few minutes of the new ultrafast sequences, to the long time required by spectroscopic imaging.

The basic multiparametric ce-MRI protocol most used includes: the non-contrast enhanced acquisitions (T2-weighted and diffusion-weighted imaging (DWI)); the native T1-weighted acquisition; and subsequently, the contrast-enhanced series. Reporting of breast MRI is standardized in the American College of Radiology Breast Imaging Reporting and Data System (BI-RADS) [18,49,50]. In good clinical practice it is important that radiologists who report a breast ce-MRI are also skilled in conventional images—mammography, ultrasound and tomosynthesis—and that since MRI often highlights lesions that are occult with conventional imaging, there is the possibility of performing MRI guided interventional maneuvers such as localization and biopsies [54].

Trying to improve lesion classification, new sequences such as DWI techniques, spectroscopic imaging and quantitative assessment of contrast material enhancement have been introduced in recent years in breast MRI. A multiparametric approach has been shown to increase the specificity of breast ce-MRI up to values equal to 90%. In particular, the use of DWI is useful to discriminate when it is necessary to perform a biopsy (ADCs greater than 1.4–3 × 10^−3^ mm^2^/sec are exceptionally rare in cancers) but also in predicting the ki67 Index in invasive ductal carcinomas [55,56]. Particular fast-field echo axial T1-weighted imaging with coronal reconstruction sequences were also evaluated for the study of axillary lymph nodes [57].

The main uses of breast MRI are: preoperative staging of breast cancer, screening of high-risk patients, evaluation during neoadjuvant chemotherapy, carcinoma of unknown primary origin (CUP syndrome) and problem solving.

In the preoperative staging of breast cancer, MRI has been shown to have a greater sensitivity than conventional imaging with values close to 100%; MRI allows a better assessment of the extent of the index lesion, with 75% of lesions differing less than one centimeter from the post-operative histology; a better assessment of the extension of the ductal component in situ associated with invasive lesions; and above all, MRI is able to identify 20% of the additional malignant lesions in the ipsilateral breast and 4–5% of additional malignant lesions in the contralateral breast. Five percent of additional malignant lesions identified by MRI are biologically more relevant than the index lesions [58]. MRI surely allows the early diagnosis of breast cancer, so why is it not always used in the preoperative staging?

The use of MRI in the preoperative staging for all patients is still much debated and the guidelines differ a lot because in the literature there are some pieces of evidence against the use of MRI. In particular, two of the three prospective trials published to date have found no improvement in surgical outcomes for patients undergoing MRI without reduction of re-excision rate, with the exception of young patients and patients with invasive lobular carcinoma [59,60]. Additionally, the meta-analyses published to date have found absolute increases in the rate of first and second line mastectomies of patients who underwent MRI compared to the group of patients who did not [61]. However, many of the studies published to date on MRI, even the prospective ones, are burdened by various limitations—in particular, by the experience of surgeons and various centers in the use of breast MRI, by the possibility of performing ultrasound second-looks and above all, by MRI guided interventions such as biopsies and localizations that allow us to make the most of the potential of the technique in the preoperative staging and the discussion of each case in the multidisciplinary meeting. This is why a prospective international multicentric study is currently underway, involving 27 centers worldwide, able to guarantee a high standard of breast MRI both in terms of equipment and in terms of workloads; the reasoning behind this is to demonstrate precisely that MRI does not increase the rate of mastectomies but rather is able to improve the surgical outcomes in all subgroups of patients [58].

**Figure 2:**
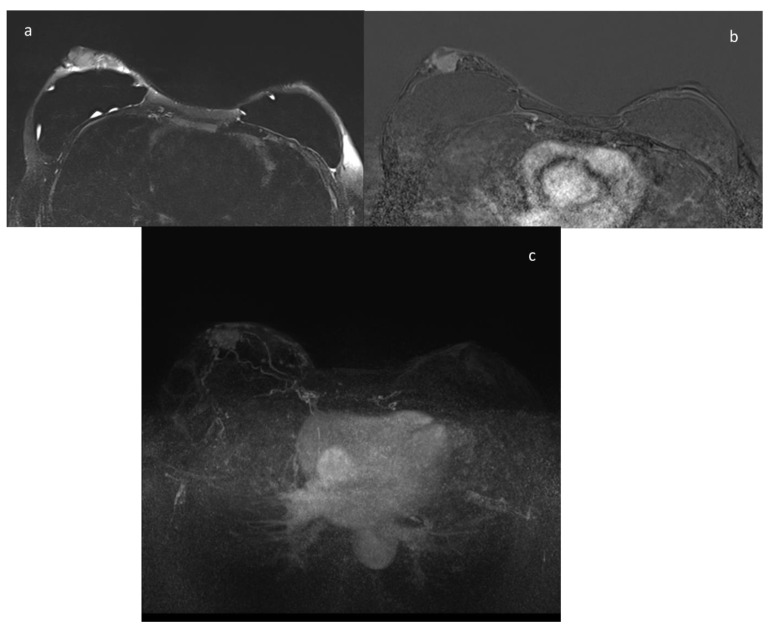
A 43-year-old patient with BRCA2 gene mutation; the patient underwent prophylactic mastectomy. At the annual ce-MRI screening a mass with irregular margins was identified in the retroareolar area of the right breast, corresponding at the US second look to a suspicious hypoechoic nodule that undergoes core needle biopsy with the diagnosis of invasive lobular carcinoma. (**a**) T2-weighted imagine; (**b**) contrast-enhanced image; (**c**) MIP image.

Ce-MRI is recommended by multiple national and international guidelines for the screening of high-risk patients as in this particular group of women, MRI can allow early diagnosis and be combined with mammography to increase the survival rates of these patients. The American Cancer Society and the American College of Radiology categorize women with a lifetime risk of more than 20% as high risk, and recommend annual screening MRI and mammography in this subset of women. This group includes patients with BRCA1 and BRCA2 gene mutations as well as other types of rarer mutations and patients who have had thoracic radiotherapy under 30, usually in the form of lymphoma therapy. Additional annual MRI screening compared to mammography alone is able to identify 4.1% more malignant lesions and to identify more aggressive invasive lesions, see Figure 2.

Using MRI for screening patients at intermediate risk, i.e., lifetime risk of between 15% and 20%, a personal history of breast cancer, dense breasts at mammography, or a history of high-risk lesions at biopsy is, on the other hand, still much debated. The studies published in the literature show that the use of MRI, also in this subgroup of patients, is able to allow earlier diagnoses compared to mammography alone with fewer false positives and a high specificity of the technique [62]. More practical limitations to the use of MRI, even in these groups of patients, are linked to the scarce availability and high costs of the technique; for this reason, the so-called abbreviated MRI was introduced (consisting only of one precontrast and one postcontrast T1-weighted acquisition) with shorter image acquisition and interpretation times that may increase the availability of breast MRI and reduce the costs [55].

In the evaluation of the residual tumor post neoadjuvant therapy, MRI has been shown to have clearly superior performance compared to conventional imaging; it is in fact able to distinguish between post-therapy fibrosis compared to the vital areas of the lesion. A recent meta-analysis including 44 studies found that the median sensitivity of MRI in detecting residual breast cancer after neoadjuvant therapy was 92% and the median specificity was 90% [63].

In the case of axillary metastasis from carcinoma of unknown primary origin with probable breast origin, MRI depicts the index lesion in the breast in 60% of cases, thereby allowing an improvement in treatment and also in the survival of patients [64].

MRI in problem solving is used for those alterations found in conventional imaging that are not certainly benign and cannot be subjected to ultrasound-guided or stereotaxical-guided needle biopsy and are based on the high negative predictive value demonstrated by MRI. In a meta-analysis, a sensitivity of 99% with an NPV of 100% was reported for the evaluation of noncalcified equivocal findings, whereas in the assessments of lesions with microcalcifications the performance of the MRI was not sufficient to avoid biopsy [65].

## 10. Contrast Enhanced Mammography (CEM)

CEM is an emerging technology. The United States (US) Food and Drug Administration’s (FDA’s) approval of the first commercial system was as recent as 2011, and this new technique is becoming more and more widespread in clinical practice and is the only alternative to MRI as a functional image. The technique with CEM is to perform a dual-energy or spectral subtraction technique. After the intravenous administration of the iodinated contrast, medium the mammography acquires in rapid succession a pair of images, first a low energy (LE) image, with the same kilovoltage and the same filter as digital mammography, and then a high-energy image that uses higher kilovoltage and an additional copper or titanium filter. Subsequently a special algorithm combines the high-energy image with the low-energy one, providing us the so called “recombined images” in which we only see the accumulation of the iodized contrast medium in the breast, and the normal tissue structures are subtracted. The images that we have at our disposal to report are the low energy image, which some published studies have shown to be similar to a conventional digital mammography and the subtracted image. This imaging method is available on several commercial mammography systems and can be performed in either 2D or 3D imaging modes.

Regarding the radiation dose administered to patients during a CEM study, the low-energy image provides the same dose as a conventional digital mammography with the high-energy acquisition of about 20%, so in total with a CEM exam the patient radiation exposure is under 1.5 times that of a normal mammographic exposure, and in any case below the limits set by international legislation [66,67]. From a recent meta-analysis published in 2018 on 84 articles, including 14,012 patients, it emerged that there is a consensus among the various authors regarding the dose of iodinated contrast medium administered, i.e., 1.5 mL/kg of body weight and on the flow rate administered, i.e., 2–5 mL/sec. The image acquisition protocol is very variable between the various centers and studies, and the authors conclude that there is a strong need to better standardize the protocol in order to be able to conduct large-scale multicenter studies in the near future, in order to obtain stronger evidence about the diagnostic performance of CEM [68].

From the data published in the literature to date we can see that the diagnostic performance of CEM is not inferior to that of MRI; in particular, the sensitivity of CEM is equivalent to that of MRI (76–100%) and seems to have even a slightly greater specificity (74–88%; see Table 2 [69,70,71,72,73,74,75,76]).

This data were further confirmed by a meta-analysis published in 2020 on 13 studies, five retrospective and eight prospective, which compared the diagnostic performance of CEM to that of MRI [77].

The most important advantages of CEM compared to MRI seem to be many: the greater availability of the technique, the greater accessibility, the higher tolerance of patients, (who in most cases prefer it to MRI), the possibility of directly viewing the microcalcifications thanks to low-energy images, the minor cost and the shorter duration time. It can also be used in patients with specific MRI contraindications, i.e., great obesity, claustrophobia, etc.; and the ease of performing second looks at suspicious areas given the patient’s position during the acquisition is more similar to that of conventional imaging than that of prone MRI. On the other hand, limitations of this new technique remain the impossibility of studying breast implants; the greater incidence of adverse reactions to iodinated contrast medium compared to gadolinium; the use of ionizing radiation, which limits the use of CEM in high-risk patients; and the impossibility, at the moment, to perform CEM-guided interventional procedures [78].

**Figure 3:**
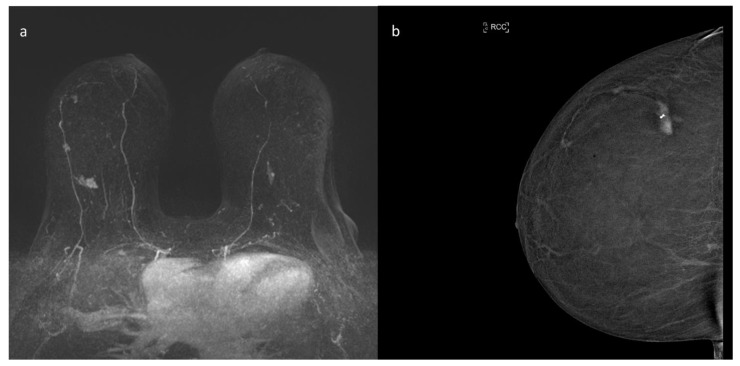
Preoperative staging with functional imaging of a 65-year-old patient with histological diagnosis of invasive multifocal ductal carcinoma of the external central quadrant of the right breast. (**a**) ce-MRI MIP image (maximum intensity projection); there are three irregular masses in the right breast referable to the multifocal index lesion. (**b**) CEM recombined images; in these images there are the three irregular masses referable to the index lesion, perfectly superimposable to that of ce-MRI.

Clinical indications of CEM seem to be similar to those of MRI with the exception of high-risk patient screening. In the preoperative staging, CEM demonstrated diagnostic performances similar to those of MRI and higher than those of conventional imaging (sensitivity ranging from 92.7 to 100% and specificity ranging from 41 to 94%), and was able to identify about 20% of additional malignant lesions in the ipsilateral or contralateral breast, thereby changing the type of surgery planned for those patients. CEM is also able to evaluate the extent of the index lesion in a way at least similar to that of MRI. CEM’s performance in the preoperative staging was very high even for invasive lobular carcinoma, the most subtle histotype of all breast carcinomas, and the greatest number of false negative results, analogously to MRI, is due to low grade and small ductal carcinomas in situ [79,80], see Figure 3.

**Figure 4:**
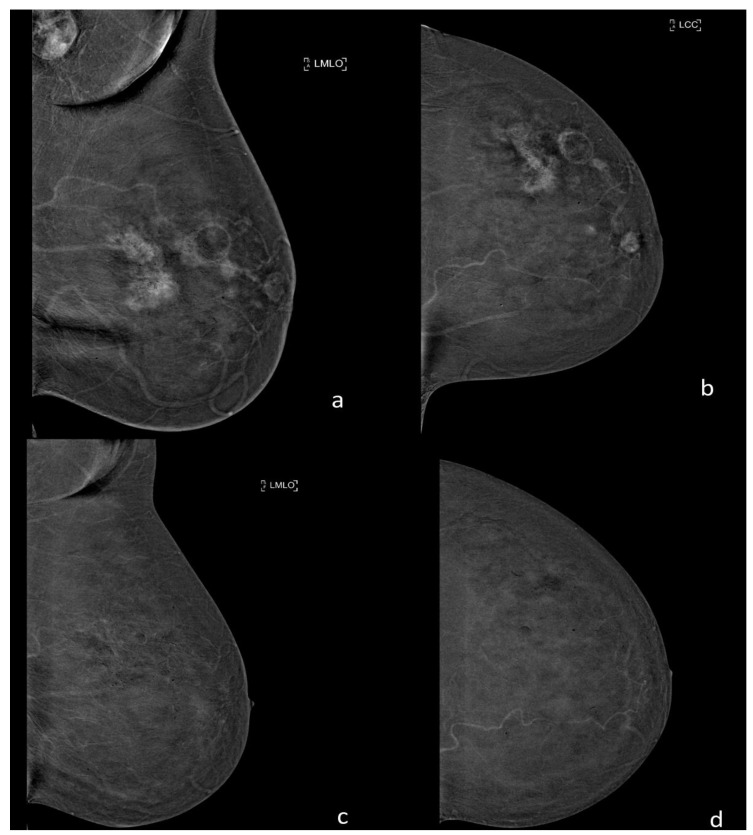
Examination with CEM before and after neoadjuvant chemotherapy of a 55-year0old patient with a multicentric invasive ductal and lobular carcinoma of the left breast. (**a**,**b**) CEM recombined images before neoadjuvant chemotherapy show multiple masses associated with an area of non-mass enhancement referable to the index lesion and a pathological lymphadenopathy in the ipsilateral axillary cavity. (**c**,**d**) CEM recombined images after neoadjuvant chemotherapy show the complete absence of pathological enhancement in the left breast, and also the regression of the axillary lymphadenopathy. The post-surgery histology confirmed the complete regression of the disease.

Additionally, with regard to the ongoing evaluation of neoadjuvant chemotherapy, CEM has shown a diagnostic performance similar to that of MRI, as confirmed by a recent meta-analysis on the topic published in 2020, which has shown values of pooled sensitivity and specificity of ce-MRI of 0.77 (95%CI, 0.67–0.84) and 0.82 (95%CI, 0.73–0.89), respectively, and values of pooled sensitivity and specificity of CESM of 0.83 (95%CI, 0.66–0.93) and 0.82 (95%CI, 0.68–0.91), respectively [81], see Figure 4.

Another very promising field of application of CEM, as demonstrated in the literature, appears to be the screening of intermediate-risk patients. In this group, CEM increased the cancer detection rate with values equal to those of MRI and superior to those of conventional imaging. CEM can also be used for screening high-risk patients who have contraindications to MRI [82].

Very promising initial results have also emerged in the application of CEM in the study of symptomatic patients, i.e., with palpable mass or nipple discharge. In this setting the sensitivity and diagnostic accuracy of CEM were clearly superior to those of DM, with a detection rate for malignancy greater than 70%, and CEM was also superior with respect to US [83].

Both in MRI and in CEM, the background parenchymal enhancement (BPE), i.e., the enhancement of the normal glandular tissue after administration of the contrast medium, is a well-known phenomenon that could make more difficult to read the images by decreasing the sensitivity of the readers. In MRI, BPE has been also shown to be an independent risk factor for breast cancer in high-risk patients. Some authors tried to correlate BPE patterns with molecular subtypes of BC, demonstrating that the BPE could help to further stratify the risk of patients who have MRIs, allowing more and more personalized screening and follow-up [84].

The future of breast imaging will certainly be closely linked to artificial intelligence, which could represent a further aid tool for early diagnosis. As already shown by applications of radiomics in CEM, which could also in this case help to better stratify the most aggressive lesions and patients at greater risk, the trend of personalization is continuing [85]. Additionally, in MRI artificial intelligence will be part of the near future, helping radiologists to better discriminate between malignant lesions and normal tissue by eliminating the BPE [86].

## 11. Conclusions

In conclusion, we have seen how functional imaging has superior diagnostic performance compared to morphological imaging and allows us to perform early diagnoses that allow continually more personalized treatments and screening for patients. MRI and CEM do not contrast with each other, but they complement each other and also integrate with conventional imaging, whose role remains indisputable, in order to always improve our clinical practice.

The main limitations, when functional imaging was constituted only by MRI, were linked to the poor availability of the technique and its high cost. Today, thanks to the introduction of CEM, this seems to be surmountable, and the functional imaging could soon become available to everyone. However, multicentric and large-scale studies are needed to better confirm the role of CEM, and to understand which patients can benefit most from functional imaging, in which situations and by which of the two techniques we have available today.

## Figures and Tables

**Table 1:** The advantage of screening mammography for mortality reduction.

Trial or Data	%
HIP RCT [15]	22
Malmo RCT [10]	22
Swedish Two-Country RCT [11]	27
Edinburgh RCT [16]	21
Stockholm RCT [15]	10
Gothenburg RCT [13]	23
Canadian service screening [17]	40
European case control studies Screened vs. not screened [14]	48

**Table 2:** Diagnostic performance of CEM compared to MRI.

Study		MRI		CEM
	Sensibility	Specificity	Sensibility	Specificity
Jochelson, Radiology, 2013 [69]	96%	n.a.	96%	n.a.
Łuczyńska, Med Sci Monit, 2015 [70]	93%	n.a.	100%	n.a.
Fallenberg, Eur Radiol, 2016 [71]	76%	88%	72%	95%
Li, Diag and Interv Imaging, 2016 [72]	100%	n.a.	100%	n.a.
Ali-Mucheru, Ann Surg Oncol, 2016 [73]	100%	n.a.	98%	n.a.
Lee-Felker, Radiology, 2017 [74]	99%	4%	94%	17%
Jochelson, Eur J of Radiol, 2017 [75]	n.a.	94.1%	n.a.	94.7%
Kim, J Breast Cancer, 2018 [76]	95.2%	73.6%	92.9%	81.1%
